# Changes in aromaticity of spin-crossover complexes: a signature for non-innocent ligands†

**DOI:** 10.1039/d3dt03404f

**Published:** 2024-01-10

**Authors:** Ana V. Cunha, Francesca Milocco, Edwin Otten, Remco W. A. Havenith

**Affiliations:** a Structural Chemistry, University of Antwerp Groenenborgerlaan 171 2020 Antwerp Belgium Ana.Cunha@uantwerpen.be; b Stratingh Institute for Chemistry and Zernike Institute for Advanced Materials, University of Groningen 9747 AG Groningen The Netherlands r.w.a.havenith@rug.nl; c Department of Chemistry, Ghent University Krijgslaan 281 (S3) B-9000 Gent Belgium

## Abstract

The influence of the spin state of the metal centre in spin crossover compounds on the aromaticity of the ligands has been investigated for iron(ii)tris-bipyridine (Fe(bpy)_3_^2+^), and Fe(ii)(formazanate)_2_ (as a truncated model and the full phenyl substituted compound). It was found that the aromaticity of the bipyridine ligands is unaffected by changing the spin state of the central iron atom, but that of the formazanate ligands is reduced upon transition to the high-spin state. This change in aromaticity is rationalized using the symmetry selection rules for aromaticity in terms of virtual excitations from occupied to empty orbitals. A further consequence of this loss in aromaticity is a shift to higher energy in the ring vibrations of the formazanate compounds that can be observed in either its IR or Raman spectrum; this prediction has been confirmed here. This change in aromaticity as a consequence of change in spin state can be regarded as an indication for non-innocent ligands.

## Abstract 2

Aromaticity of non-innocent ligands is affected when the spin state changes.

## Graphical abstract

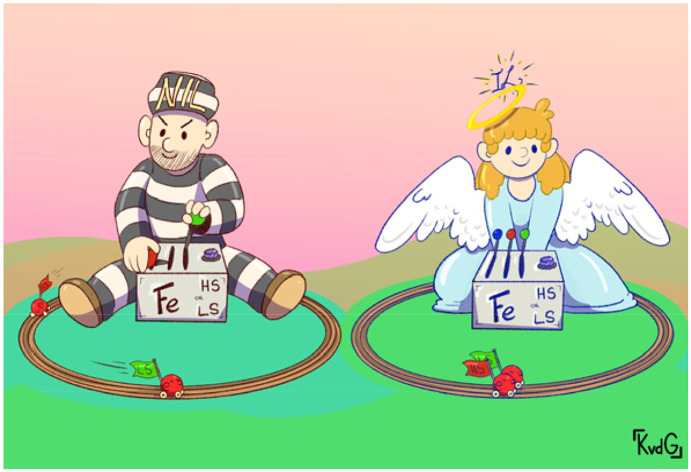

## Introduction

Materials with spin switching behaviour are of interest for their potential application in electronic devices. Spin-crossover compounds also show different chemical reactivity in their different spin states, and changes between potential energy surfaces with different spin state are key to the reactivity of both synthetic catalysts as well as enzymes (‘two-state reactivity’).^1–3^ Although the spin-crossover phenomenon was recognized almost a century ago,^4^ a more general understanding and appreciation of its relevance was developed much later.^5,6^ Many spin-crossover compounds are based on six coordinated transition metal compounds,^7,8^ and the energies between the d-orbitals determine the spin-crossover behavior. These orbital energies can be manipulated by exploiting ligand effects, thereby influencing the relative energies between the spin states.^9^ The most commonly studied spin crossover complexes are Fe(ii) complexes with a d^6^ configuration, octahedrally coordinated by nitrogen-based ligands.^10^ The spin-crossover behaviour is affected at a molecular level by changes in the steric and electronic properties of the ligands. In addition, the energetics of spin-crossover in the solid state are influenced by packing and other intermolecular forces. These can lead to cooperative effects resulting in thermal hysteresis, which is important for memory applications.^11,12^

Four coordinated transition metal compounds usually have a high-spin ground state, due to the smaller d-orbital energy splittings. Nevertheless, also in these compounds it is possible to provide spin crossover behavior by judicious ligand design as first demonstrated by Smith and co-workers.^13^ A four-coordinated Fe(ii) spin-crossover complex with formazanate ligands has been reported; the study revealed that this compound possesses an unusually stable low-spin state.^14^ It was shown that this stabilization of the low-spin state originates from an “inverted” ligand field^15^ that is due to the π-acceptor properties of the ligand which stabilizes one of the d-orbitals that is normally antibonding. This back-donation from the metal to the formazanate ligands was corroborated by an intrinsic bond orbital (IBO) analysis. Three occupied IBOs that have d-character at the iron centre were found for low-spin iron(ii)bis(formazanate): one of these was almost purely localized on the Fe(ii) centre, while the other two IBOs were found to be π-bonding with the ligand, shown by substantial delocalization onto the formazanate N atoms. This further indicates that the formazanate ligand is not a mere spectator ligand, but it can be classified as ‘noninnocent’.^15–23^ Similar low-coordinate metal complexes with noninnocent ligands are important from the perspective of catalysis, and thus an understanding of how the difference in energy between the spin states can be altered is desirable.

**Fig. 1:**
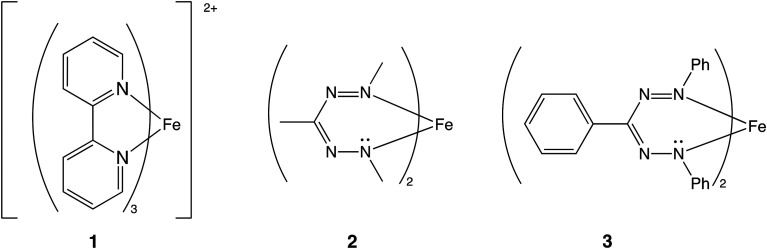
The molecules under study.

In this contribution, we investigate whether the spin state of the transition metal influences the aromaticity of the ligands, and if the change in aromaticity in the ligands can play a role in determining the spin state energy differences. We have studied the compounds computationally, and have verified our predictions experimentally using IR spectroscopy. The systems that we study are iron(ii)tris-bipyridine (Fe(bpy)_3_^2+^, 1) and Fe(ii)(formazanate)_2_, both as a truncated model (2) and the full system studied experimentally (3, Fig. 1). Compound 1 was chosen as it is a well-known spin crossover compound,^24^ with innocent ligands, while the ligands of 2 and 3 are classified as non-innocent.^14,25^

To assess the aromaticity of the ligands, we calculate the magnetically induced current density using the ipsocentric Continuous Transformation of Origin of Current Density – Diamagnetic Zero (CTOCD-DZ) formulation.^26–28^ In this formulation, the induced current density can be decomposed into orbital contributions (be it canonical or localized orbitals^29^), and the magnetic response can be further interpreted in terms of virtual transitions from occupied to empty orbitals.^30–32^ These virtual transitions that give rise to diatropic (aromatic) contributions to the induced current are governed by the linear momentum operator, while those that contribute to paratropic (antiaromatic) currents are governed by the angular momentum operator.

## Computational methods

Geometries of singlet, triplet, and quintet 1, 2 and 3 were optimized using AMS2022.^33–35^ The PBE functional was chosen and the TZ2P basis set (no frozen core) was used. The stationary points were characterized as minima through calculation of the frequencies, leading also to the simulated IR spectra (with line broadening of 50 cm^−1^). The optimized geometries are in good agreement with previously published results.^14,36^ The ring current calculations were performed using the CTOCD-DZ method^27,28,30,31,37^ as implemented in GAMESS-UK^38,39^ and SYSMO^40^ at the PBE/def2-SVP level, taken from the Basis Set Exchange Library.^41–43^ The def2-SVP basis set is sufficient for the calculation of ring current plots, as previous studies have shown that these plots converge quickly with basis set size.^44^ As previous studies have shown, the ring current patterns are determined by the nodal structure of the frontier orbitals,^30,31^ hence, the current density plots do not depend heavily on choice of functional.^39,45^ This is also verified for 2, for which we have recalculated the π-current density plots using the PBE0 functional (Fig. S2†), which are visually identical to the PBE ones. For the open-shell species UPBE was used, following established protocols published in the literature.^46,47^ The ring current is plotted in a plane 1 *a*_0_ above one of the aromatic rings. In the plots, diatropic (paratropic) current is anticlockwise (clockwise). The localized π-like orbitals were obtained after a Pipek–Mezey localization,^48^ and their contribution to the current density was calculated using the procedure outlined in reference.^29^ Details on the IR spectroscopic measurements can be found in the ESI.†

## Results and discussion

The energies of the different spin states together with the Mulliken spin population at the Fe centre, and averaged Fe–N bond lengths are listed in Table 1. The energy and Gibbs free energy differences evaluated with the PBE functional for 1 for the quintet states are in line with the calculated CASPT2 value of 0.69 eV.^36^ However, in our calculations, the triplet is in between the singlet and quintet, while the reported CASPT2 calculations predict the triplet state to be above the quintet state. Although the PBE energies may not be the most accurate ones, the PBE trend that the singlet state is the lowest state for all three compounds is in agreement with experiment.^14,49^ For the (truncated) formazanate complexes 2 and 3, the calculated Fe–N bond lengths also agree with experimental data and with previously reported results; in the singlet state, the structure of 2/3 can be described as a flattened D_2d_ structure, with an angle between the two Fe(NNNN) planes of *ca.* 70°, that increases to *ca.* 98° (2)/82° (3) in the quintet state. For both compounds, shorter Fe–N bond lengths are found for the singlet spin state, while for the high-spin state, longer Fe–N bond distances are found, due to occupation of the antibonding e_g_ orbitals. For 1, no further significant changes are observed in the geometry of the ligands.

**Table 1:** Relative (PBE) energy (eV) of the different spin states together with the Mulliken and MDC^50^ (in parentheses) spin populations (PBE) on the Fe-centre and the ligands for the different spin states of 1, 2, and 3

	Δ*E*	Δ*G*	Spin pop-Fe	Spin pop-ligands	Fe–N	Fe–N (exp.)
1-Singlet	0.00	0.00	—	—	1.97	1.97^49^
1-Triplet	1.13	0.97	2.087 (1.986)	−0.087 (0.014)	1.97–2.24	—
1-Quintet	1.33	1.09	3.766 (3.600)	0.238 (0.400)	2.18	2.19^a^
2-Singlet	0.00	0.00	—	—	1.82	1.83^14^
2-Triplet	0.61	0.52	2.011 (1.836)	−0.011 (0.164)	1.88	—
2-Quintet	1.16	0.84	3.640 (3.399)	0.360 (0.601)	1.96	1.97^14^
3-Singlet	0.00	0.00	—	—	1.83	1.83^14^
3-Quintet	0.68	0.53	3.674 (3.411)	0.326 (0.589)	1.99	1.97^14^

a Calculated value.^36^

**Fig. 2:**
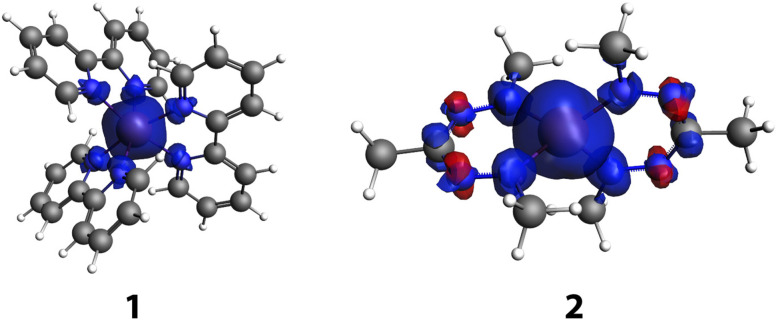
Isosurface (isovalue 0.003) plot of the spin density in the quintet states of 1 and 2.

For both compounds, some spin delocalization is discernible, especially for the quintet state. For 2, both the Mulliken and multipole derived (MDC)^50^ spin populations are slightly larger on the ligands than for 1, but no marked differences are found for the spectator ligands of 1 compared to the non-innocent ligands of 2. However, inspection of the spin density plots (Fig. 2) shows a marked difference between the two: for 1, spin density is found at the nitrogen atoms that coordinate to the iron atom, whereas for 2, spin density and spin polarization is also found in the π system of the ligand. This may already hint at the difference in behavior of the two ligands. Compound 3 shows similar spin populations on the iron centre and ligands in the quintet state as 2.

**Fig. 3:**
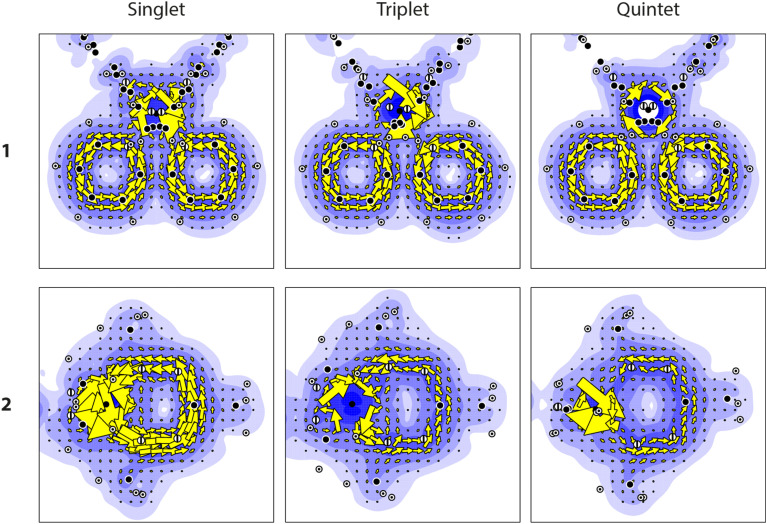
The d + π induced current density for 1 and 2 in their singlet, triplet, and quintet state.

Now turning to the aromaticity of the ligands by inspection of the induced current density. The sum of the contributions of the localized π and Fe-d orbitals to the induced current density is plotted in Fig. 3 for the different spin states. For 1, for all spin states, a diatropic current is visible in the bipyridine ligands. This ring current is unperturbed by the Fe atom, and is unaffected by the spin state of the Fe atom. The invariance of the ring current is further corroborated by the maximum of the current density, *j*_max_ (Table 2), which is identical for all three spin states. The aromatic character of the bipyridine ligands in 1 is virtually unaffected by the spin state of the Fe atom, and, thus rightfully, the bipyridine ligand can be classified as a spectator ligand in this system.

**Table 2:** The *j*_max_ values (au) for the d + π induced current densities in the different spin states of 1 and 2

Compound	Singlet	Triplet	Quintet
**1**	0.064	0.066	0.062
2	0.103	0.054	0.050

The situation for 2 is rather different: although for all three spin states, a diatropic current is observed in the formazanate ligand, the strength of this current is changed significantly. For the singlet case, a strong current is found, while the current for both the triplet and quintet is notably weaker. This observation is corroborated by the maximum strength of induced current density, *j*_max_, as well: *j*_max_ is almost reduced by a factor of 2 when the spin state of the Fe atom changes from singlet to triplet. However, when the spin state changes from triplet to quintet, no appreciable change in the current density and *j*_max_ is found (Table 2).

**Fig. 4:**
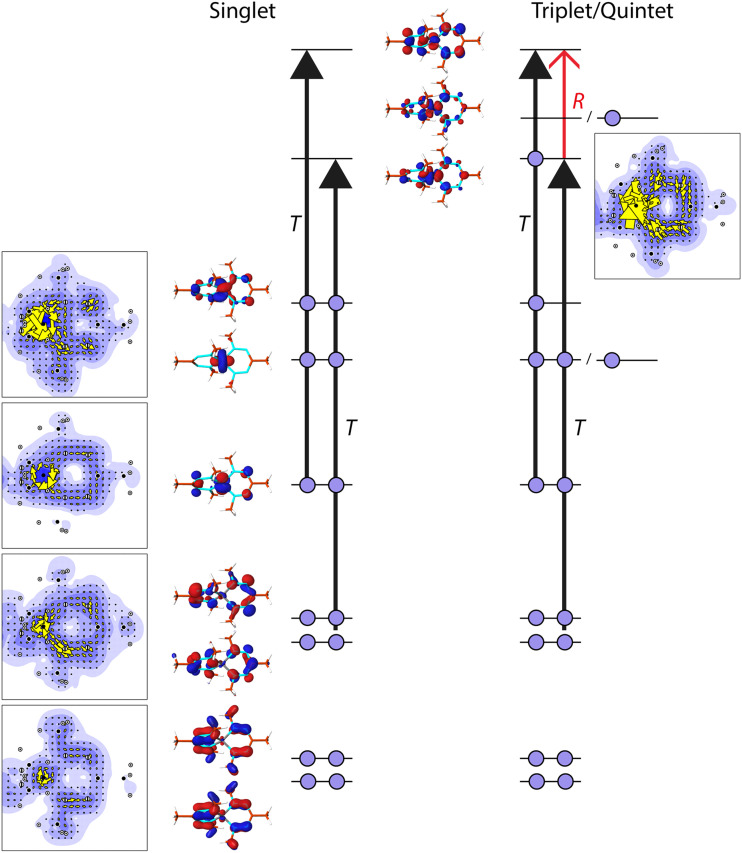
The orbital contributions to the π ring current for 2 in the singlet and triplet/quintet states. Indicated are the main virtual transitions, responsible for the diatropic ring current in singlet-2 and the reduced diatropic ring current in triplet/quintet-2. *T* indicates a translationally allowed (contributing to the diatropic current density) transition, while *R* indicates a rotationally allowed (contributing to the paratropic current density) transition.

The observed differences in current density between 1 and 2 and their different spin states can be explained within the ipsocentric model by considering which localized orbitals and virtual excitations govern the ring current pattern (Fig. 4). In the singlet state of 2, the three t_2g_-like d-orbitals are doubly occupied, together with six electrons in the π-like system of the formazanate ligand. The lowest π-orbital has hardly any contribution to the ring current, while the two higher lying π-orbitals contribute in a diatropic fashion to it. More strikingly, one of the d-orbitals also contributes diatropically to the π-ring current. The other two occupied d-orbitals do not contribute to the ring current, but they show paratropic, local circulations around the Fe-centre. Thus, the π system of the formazanate ligand consists of eight π-electrons, while the ring current is dominated by the contributions of three doubly occupied orbitals. The d-orbital of the Fe-centre actively participates in the ring current, leading to a Möbius like orbital structure (a cyclic array of orbitals with an odd number of out-of-phase overlaps) and Möbius aromaticity.^51–53^ The excitations that dominate these contributions are the translationally allowed transitions (T) from the occupied π-orbitals to the anti-bonding, empty, π-orbitals (Fig. 4).

To form the triplet state of 2, one electron from one of the β-d-orbitals that does not contribute to the ring current is excited into the α-π-anti-bonding orbital (Fig. 4). The consequence of this excitation is that a new virtual transition is allowed, *viz.* from the π-orbital that is newly occupied to the other remaining empty π-orbital. This transition is allowed for a rotational transition (R), thus *via* the angular momentum operator, and it will have a paratropic contribution. Indeed, the contribution from this orbital is found to be paratropic (Fig. 4). The allowed transitions that govern the ring current in the β-spin manifold remains unchanged.

To form the quintet state, one β-electron from the other, doubly occupied, spectator, σ-like, d-orbital is excited to the last remaining empty α-(σ-like-)d-orbital. Hence, no changes occur in occupations in the π-like orbitals, and consequently, the allowed excitations that give rise to the π ring current pattern in the quintet state are identical to those in the triplet state. It is thus expected that the ring currents for the triplet and quintet states are similar, as is observed (Fig. 3).

**Fig. 5:**
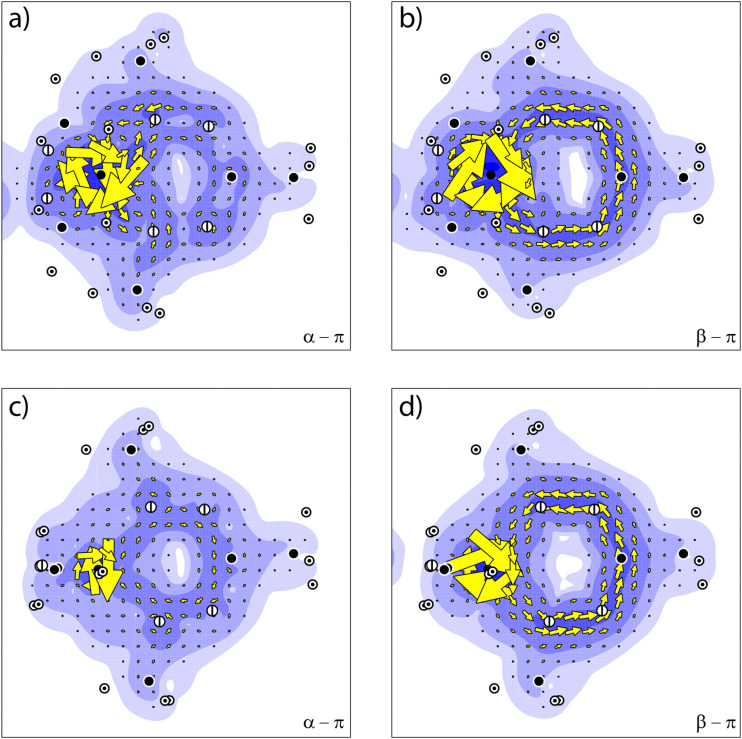
The ring current for 2 of (a) the α-π orbitals occupied in the triplet state, (b) the β-π orbitals occupied in the triplet state, (c) the α-π orbitals occupied in the quintet state, and (d) the β-π orbitals occupied in the quintet state.

The sum of the orbital contributions for the d- and π-orbitals is plotted for the different spin manifolds for triplet and quintet 2 in Fig. 5. The plots confirm the ring current patterns that have been deduced from a consideration of the allowed virtual transitions: the diatropic ring current in the α spin manifold is quenched, due to the paratropic contribution of the α-d_π_ orbital, whereas the ring current in the β-spin manifold remains diatropic. Furthermore, these contributions are for the quintet state of 2 indistinguishable to those for the triplet state. This is not unexpected as in the quintet state, the d-orbitals that do not participate in the π-system become occupied.

The situation for 2 is substantially different from the situation in 1: in 1, the d-orbitals are mainly localized on the Fe-centre, and do not mix with the π-system of the ligands. In this sense, the ligands are mere spectator ligands. Thus the π-system of the ligands is hardly affected by changes in occupation of the d-orbitals, leaving the allowed virtual transitions, and thus the ring current, unchanged. In contrast in 2, the d-orbitals actively mix with the π-orbitals of the ligands, thus changes in the occupations of the d-orbitals directly influence the allowed transitions in the π-system. This mixing of the d-orbitals with the π-orbitals of the ligands is reminiscent for the noninnocence of the formazanate ligand, and changes in the ring current as a consequence of changes in the spin state of the central metal can be regarded as a sign for the noninnocence of the ligands.

A remaining question that needs to be addressed is whether this change in aromaticity due to changes in the spin state can be measured experimentally. One particular IR mode of b_2u_ symmetry in benzene, the ‘Kekulé’-mode that interconverts the two equivalent D_3h_ Kekulé structures, shifts to higher energy in the ^1^B_2u_ excited state of benzene: its IR frequency shifts 261 cm^−1^ to higher energy in the excited state.^54,55^ This shift has been connected to the tendency of the π-electrons to distort. Hence, if the aromaticity changes, a change in this mode is expected.

The IR frequencies of the ‘Kekulé’-modes in 1 and 2 have been calculated (Table 3) for the different spin states. For 1, where there is no change in aromaticity in the bipyridine ligands, no significant changes are predicted by these calculations. However, for 2, the frequency shifts by almost 50 cm^−1^ to higher energy when going from the singlet to the triplet state, which supports the decrease in aromaticity of the triplet state. Increasing the spin state further to the quintet state does not lead to an additional shift in the IR frequency, which supports the observation that the aromaticity does not change further when going from the triplet to the quintet state.

**Table 3:** Calculated IR frequencies of the ‘D_6h_→D_3h_’ vibrational modes (cm^−1^) for 1 and 2, and their shift with respect to the singlet state in parentheses

Compound	Singlet	Triplet	Quintet
**1**	1290/1300	1285/1302 (−5/+2)	1289/1306 (−1/+6)
**2**	1207/1228	1250/1265 (+43/+37)	1253/1255 (+46/+27)

**Fig. 6:**
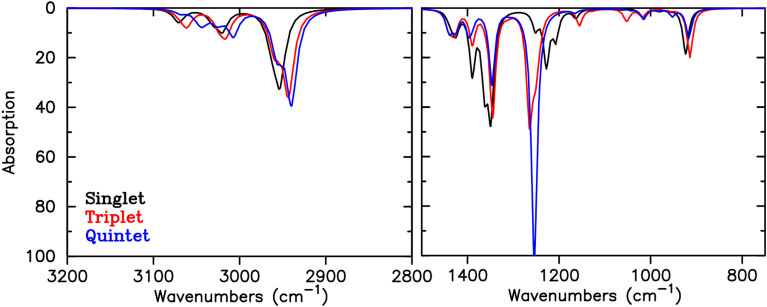
Calculated IR spectrum for the different spin states of 2.

The calculated IR spectra for the different spin states of 2 (Fig. 6) show some further changes besides the frequency shift of the ‘Kekulé’-modes that are expected upon changing spin state: the intensity of the peaks related to the ‘Kekulé’-modes increases upon increase in spin state. For 1, only changes in intensities in the IR spectra are calculated (Fig. S1†), consistent with no appreciable changes in aromaticity. The peaks that show the largest changes in intensity in the IR spectrum of 1 are related to other vibrations than the ‘Kekulé’-modes.

**Fig. 7:**
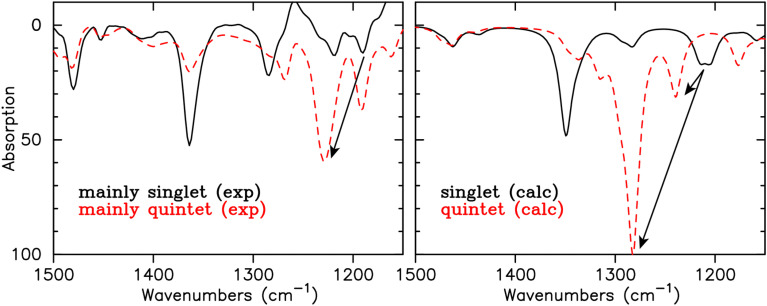
Experimental (left) and calculated (right) IR spectrum for singlet and quintet 3. Indicated with the arrow is the shift in frequency for the ring vibrations upon transition to the quintet state.

To verify this prediction, we also calculated and measured the IR spectrum (Fig. 7) of the experimentally available phenyl substituted analogue of 2, *viz.*3 (Fig. 1). For 3, the Kekulé-mode is not a ‘pure’ vibration mode of the six-membered ring of interest, due to coupling with the modes in the phenyl substituents. Nevertheless, clear Fe(N–N–CR–N–N) ring vibrations are discernible at 1202 and 1214 cm^−1^ for the singlet state, that are shifted to 1239 and 1279/1281 cm^−1^ in the quintet state. This shift is indicative for loss of aromaticity in the Fe-formazanate ring. In the IR spectrum of 3, the shift to higher energy is accompanied with an increase in intensity, and we anticipated that this distinct change in the IR spectrum should be experimentally discernable. To further corroborate our predictions, we performed variable-temperature IR spectroscopy using a 50 mM solution of 3 in anhydrous THF. Spectra were measured using a diamond probe directly inserted into the analyte solution; the probe was connected *via* a fiber conduit to a liquid N_2_-cooled MCT detector. The flask containing 3 in THF was immersed in a cooling/heating bath, and its internal temperature was recorded with the integrated sensor of the IR probe. Spectra of pure THF were collected at various temperatures and subsequently used for solvent subtraction to provide the IR spectrum of 3 at temperatures between −45 and +53 °C. Although the solvent absorbs strongly in the 800–950 and 1000–1100 cm^−1^ range, which causes some artefacts upon solvent subtraction, the absorption bands of interest are sufficiently separated (>1150 cm^−1^) to be useful for our analysis. The changes observed in the IR spectra upon cooling/heating are fully reversible, which confirms that these are related to the spin-state equilibrium in 3 rather than decomposition. At the extremes of the temperature range examined, the LS : HS ratio is calculated to be ∼95 : 5 (−45 °C) and ∼52 : 48 (53 °C) based on the thermodynamic equilibrium parameters measured previously (Δ*H* = 17.9 ± 0.1 kJ mol^−1^ and Δ*S* = 54 ± 1 J mol^−1^ K^−1^).^14^ One of the absorption bands around 1200 shows a marked shift to higher wavenumber (1229 cm^−1^) upon increasing the temperature, with a concomitant increase in intensity. The decrease in the intensity for the band at 1364 cm^−1^, predicted by the calculations, is also clearly visible.

## Conclusions

For non-innocent ligands, the metal centred d-orbitals mix with the π-system of the ligands, and, hence, changes in spin state of the metal centre directly influences the allowed virtual transitions that govern the π ring current in the ligands. In iron(ii)formazanate, a Möbius aromatic singlet state is found. Upon spin crossover to the quintet (or triplet) state, the aromaticity in the α spin manifold is quenched, following the ipsocentric selection rules for ring current, due to the rotationally allowed virtual transition that has become available as a result from this excitation. The aromaticity of the β spin manifold is unaffected by the change in spin state. For spectator ligands, such as the bipyridine ligand in 1, changes in the spin state of the metal centre are not reflected in changes in ring current, as the d-orbitals of the metal centre do not actively participate in the π-system of the ligands. Hence, changes in aromaticity, as probed by the induced current density, due to changes in spin state of the central metal centre is a signature for the non-innocence of the ligands. This decrease in aromaticity is further confirmed by the IR spectrum for the low and high-spin states of 3.

## Conflicts of interest

There are no conflicts to declare.

## Supplementary Material

DT-053-D3DT03404F-s001
