# Supplementary material for: Changes in aromaticity of spin-crossover complexes: a signature for non-innocent ligands
Source: Dalton Trans. 2024 Jan 10;53(6):2789–96. doi: 10.1039/d3dt03404f (PMC10845013; doi:10.1039/d3dt03404f)
Supplement: DT-053-D3DT03404F-s001 [file DT-053-D3DT03404F-s001.pdf]

Electronic Supplementary Material (ESI) for Dalton Transactions.
This journal is © The Royal Society of Chemistry 2024

Supporting Information

by

Changes in aromaticity of spin-crossover complexes: a signature for non-innocent ligands

Ana V. Cunha,*[a] Francesca Milocco,[b] Edwin Otten,[b] and Remco W.A. Havenith*[b,c]

[a]Structural Chemistry, University of Antwerp, Groenenborgerlaan 171, 2020 Antwerp, Belgium

[b]Stratingh Institute for Chemistry and Zernike Institute for Advanced Materials, University of Groningen, 9747 AG Groningen, The Netherlands

[c]Department of Chemistry, Ghent University, Krijgslaan 281 (S3), B-9000 Gent, Belgium

Table of Contents

Experimental details	S2
Table S1. Cartesian coordinates of singlet **1**	S3
Table S2. Cartesian coordinates of triplet **1**	S4
Table S3. Cartesian coordinates of quintet **1**	S6
Table S4. Cartesian coordinates of singlet **2**	S7
Table S5. Cartesian coordinates of triplet **2**	S8
Table S6. Cartesian coordinates of quintet **2**	S9
Table S7. Cartesian coordinates of singlet **3**	S10
Table S8. Cartesian coordinates of quintet **3**	S12
Figure S1. Calculated IR spectrum for the different spin states of **1**	S14
Figure S2. Plots of the induced current π-density using PBE0 for the different spin states of **2**	S14

Experimental details

All manipulations were carried out under nitrogen using standard glovebox and Schlenk-line techniques. THF was dried by percolation over a column of Al_2O_3 (Fluka). Compound **3** was prepared as described in the literature.[1]
Solution IR spectra were acquired using a Mettler Toledo ReactIR 700 instrument equipped with a DiComp (diamond) probe and a AgX Fiber Conduit connected to a liquid N_2-cooled MCT detector. Spectra were recorded with 4 nm^{-1} resolution. The temperature of the solution was controlled by immersing the flask in an ethanol bath (for temperatures below room temperature). The bath temperature was regulated using a Julabo FT902 immersion chiller, and the internal temperature of the solution of **3** was monitored using the integrated temperature sensor in the IR probe. For temperatures above room temperature, the flask was immersed in an oil bath that was heated to the desired temperature on a hotplate. A Schlenk flask with a 50 mM solution of **3** in anhydrous THF was prepared inside a glovebox, and subsequently transferred to the ReactIR setup. Under a counterflow of N_2, the probhead was inserted into the flask.
The IR measurements were started around 15 °C, and the flask was subsequently cooled to -45 °C while continuously measuring the IR spectrum (every ~10 seconds). The spectra were analyzed using *iC IR* 7.1.
The experiment was repeated but then with only pure THF to collect IR spectra for background subtraction. Spectra recorded at selected temperatures were imported into *Spectragryph-on* 1.2.16, and after base-line correction the appropriate THF spectrum was subtracted from that of **3**/THF collected at the same temperature.

The largest changes are observed for the spectra at the extremes of the temperature range (in this case -45 and + 53 °C); these are used for the discussion in the main text.

1. Milocco, F.; de Vries, F.; Bartels, I. M. A.; Havenith, R. W. A.; Cirera, J.; Demeshko, S.; Meyer, F.; Otten, E., Electronic control of spin-crossover properties in four-coordinate bis(formazanate) iron(II) complexes. *J. Am. Chem. Soc.* **2020,** *142*, 20170-20181 (ref 14 of maintext).

Table S1. Cartesian coordinates of PBE/TZ2P optimized singlet **1** (Å).

Atom	X	Y	Z
Fe	-0.000635	-0.009625	-0.003364
N	-0.424316	1.916959	-0.008296
N	1.841034	0.696780	-0.001423
N	0.538841	-1.901778	0.121700
N	-1.879528	-0.597147	-0.122125
N	0.105165	-0.154714	1.960717
N	-0.185680	-0.004935	-1.966952
C	0.657854	2.755098	-0.021141
C	1.945757	2.061207	0.023688
C	0.506979	4.144713	-0.071817
C	3.189045	2.699197	0.085885
C	-0.766990	4.701041	-0.108436
C	4.354127	1.940542	0.118530
C	-1.868509	3.844824	-0.093289
C	4.244836	0.549907	0.088862
C	-1.657556	2.471797	-0.045543
C	2.982469	-0.028799	0.030466
C	0.705436	-2.380837	1.393128
C	-2.379052	-0.709522	-1.391733
C	1.044544	-3.716292	1.633283
C	-3.685577	-1.149546	-1.627909
C	1.218366	-4.588214	0.564005
C	-4.505872	-1.486714	-0.556522
C	1.044727	-4.098053	-0.730928
C	-3.992787	-1.375262	0.736232
C	0.707305	-2.761456	-0.908972
C	-2.686662	-0.932314	0.910440
C	0.494336	-1.377356	2.437424
C	-1.427077	-0.335784	-2.439170
C	0.662438	-1.609608	3.806497
C	-1.719858	-0.313134	-3.806671
C	0.431036	-0.582507	4.715197
C	-0.733578	0.046904	-4.718739
C	0.033581	0.662638	4.226921
C	0.532591	0.378054	-4.235123
C	-0.114922	0.835721	2.855648
C	0.764265	0.342747	-2.865218
H	1.384793	4.789346	-0.085699
H	3.244570	3.786618	0.110923
H	-0.898511	5.782606	-0.147098
H	5.329455	2.425224	0.166638

H	-2.887961	4.229447	-0.118123
H	5.126840	-0.090009	0.110392
H	-2.499349	1.782852	-0.029774
H	2.869037	-1.110571	0.005504
H	1.168395	-4.072840	2.654964
H	-4.057645	-1.232507	-2.648168
H	1.482498	-5.631597	0.737450
H	-5.526346	-1.830616	-0.726838
H	1.168240	-4.740135	-1.602822
H	-4.593493	-1.628032	1.609769
H	0.569006	-2.352540	-1.907787
H	-2.263753	-0.832497	1.907876
H	0.976925	-2.590830	4.159317
H	-2.717950	-0.573987	-4.155986
H	0.557517	-0.750288	5.784988
H	-0.948329	0.068181	-5.787324
H	-0.162181	1.499589	4.896961
H	1.341030	0.664141	-4.907676
H	-0.425681	1.795648	2.448656
H	1.742305	0.596042	-2.461393

Table S2. Cartesian coordinates of PBE/TZ2P optimized triplet **1** (Å).

Atom	X	Y	Z
Fe	-0.000074	0.012948	-0.019072
N	1.241203	-0.810985	1.663235
N	-0.917036	0.742358	1.612212
N	-1.220215	1.058184	-1.573329
N	1.003822	-0.907554	-1.453162
N	0.927838	1.794781	-0.195647
N	-1.008162	-1.694873	-0.058473
C	0.658072	-0.588010	2.868018
C	-0.470634	0.363902	2.848211
C	1.108975	-1.230192	4.029037
C	-1.058819	0.877779	4.010684
C	2.190588	-2.102843	3.948268
C	-2.116911	1.775479	3.920134
C	2.795951	-2.318660	2.709570
C	-2.567155	2.154489	2.655187
C	2.284010	-1.657933	1.596239
C	-1.939972	1.624010	1.535088
C	-0.666039	2.238379	-1.950128
C	0.593920	-2.184952	-1.738219

C	-1.141835	2.948581	-3.060391
C	1.248246	-2.961322	-2.700859
C	-2.217075	2.441374	-3.784301
C	2.337043	-2.437454	-3.387424
C	-2.790477	1.232424	-3.387418
C	2.751892	-1.135888	-3.094546
C	-2.255289	0.574158	-2.283539
C	2.064984	-0.409129	-2.131441
C	0.458616	2.695209	-1.110479
C	-0.558767	-2.624685	-0.959995
C	1.021949	3.973269	-1.213003
C	-1.182598	-3.870143	-1.093550
C	2.079781	4.338152	-0.387849
C	-2.285597	-4.177763	-0.306142
C	2.558514	3.409606	0.537011
C	-2.746700	-3.224313	0.604938
C	1.954259	2.160812	0.605175
C	-2.085859	-2.007141	0.700200
H	0.618677	-1.062229	4.986881
H	-0.678518	0.584299	4.987831
H	2.553832	-2.611455	4.841965
H	-2.578604	2.177468	4.822402
H	3.648881	-2.988907	2.603529
H	-3.391822	2.855793	2.529626
H	2.725969	-1.803528	0.608353
H	-2.260130	1.894189	0.529696
H	-0.674335	3.883315	-3.366731
H	0.906522	-3.974173	-2.909798
H	-2.600113	2.982010	-4.650459
H	2.854702	-3.033336	-4.139473
H	-3.636118	0.802008	-3.923758
H	3.599149	-0.681048	-3.607360
H	-2.671375	-0.376933	-1.945017
H	2.363012	0.607704	-1.882928
H	0.621429	4.687969	-1.930113
H	-0.807194	-4.594660	-1.814804
H	2.519989	5.332924	-0.461727
H	-2.779668	-5.145034	-0.400593
H	3.386200	3.645157	1.205624
H	-3.609035	-3.418307	1.242461
H	2.293922	1.408997	1.316327
H	-2.419582	-1.246909	1.403823

Table S3. Cartesian coordinates of PBE/TZ2P optimized quintet **1** (Å).

Atom	X	Y	Z
Fe	0.000000	0.000000	-0.028250
N	-1.326566	0.113419	1.686576
N	1.326566	-0.113419	1.686576
N	1.542757	-0.460440	-1.503560
N	-1.542757	0.460440	-1.503560
N	-0.042744	-2.189129	-0.205889
N	0.042744	2.189129	-0.205889
C	-0.733424	0.086728	2.911922
C	0.733424	-0.086728	2.911922
C	-1.489264	0.223444	4.084146
C	1.489264	-0.223444	4.084146
C	-2.868883	0.379951	4.003358
C	2.868883	-0.379951	4.003358
C	-3.472100	0.401195	2.745012
C	3.472100	-0.401195	2.745012
C	-2.665184	0.269473	1.620896
C	2.665184	-0.269473	1.620896
C	1.671909	-1.769549	-1.846165
C	-1.671909	1.769549	-1.846165
C	2.492528	-2.156447	-2.912876
C	-2.492528	2.156447	-2.912876
C	3.197770	-1.189724	-3.625062
C	-3.197770	1.189724	-3.625062
C	3.065598	0.150999	-3.263055
C	-3.065598	-0.150999	-3.263055
C	2.224780	0.468285	-2.201218
C	-2.224780	-0.468285	-2.201218
C	0.881603	-2.732814	-1.042588
C	-0.881603	2.732814	-1.042588
C	1.063023	-4.118755	-1.123622
C	-1.063023	4.118755	-1.123622
C	0.266409	-4.962991	-0.354921
C	-0.266409	4.962991	-0.354921
C	-0.692923	-4.401989	0.487672
C	0.692923	4.401989	0.487672
C	-0.803825	-3.015587	0.537071
C	0.803825	3.015587	0.537071
H	-1.005341	0.213903	5.059208
H	1.005341	-0.213903	5.059208
H	-3.464640	0.486701	4.910445
H	3.464640	-0.486701	4.910445

H	-4.549324	0.521500	2.631500
H	4.549324	-0.521500	2.631500
H	-3.097286	0.288197	0.619532
H	3.097286	-0.288197	0.619532
H	2.573004	-3.204007	-3.198678
H	-2.573004	3.204007	-3.198678
H	3.839059	-1.481319	-4.457724
H	-3.839059	1.481319	-4.457724
H	3.600254	0.939712	-3.791782
H	-3.600254	-0.939712	-3.791782
H	2.086338	1.503410	-1.884428
H	-2.086338	-1.503410	-1.884428
H	1.828035	-4.541134	-1.773243
H	-1.828035	4.541134	-1.773243
H	0.397452	-6.044240	-0.410134
H	-0.397452	6.044240	-0.410134
H	-1.340878	-5.023175	1.105811
H	1.340878	5.023175	1.105811
H	-1.529382	-2.536917	1.196757
H	1.529382	2.536917	1.196757

Table S4. Cartesian coordinates of PBE/TZ2P optimized singlet **2** (Å).

Atom	X	Y	Z
Fe	-0.000684	-0.028857	-0.005702
N	1.276153	0.294306	1.244048
N	2.588232	0.144707	1.183024
N	1.266889	-0.381196	-1.256891
N	2.580969	-0.260887	-1.197607
N	-1.256209	1.265545	0.198792
N	-2.570413	1.207693	0.075544
N	-1.288136	-1.292552	-0.207869
N	-2.600183	-1.202307	-0.076750
C	3.172654	-0.062840	-0.008254
C	4.677686	-0.051467	-0.016725
C	0.851083	0.535293	2.635878
C	-0.874408	-2.706328	-0.288792
C	0.834184	-0.617618	-2.647370
C	-3.173652	0.010096	-0.002054
C	-0.807741	2.668133	0.288688
C	-4.678111	0.030572	-0.033980
H	1.373173	1.418857	3.031661
H	1.109763	-0.328711	3.264741

H	-0.231721	0.696549	2.658985
H	0.276012	2.690310	0.443354
H	-1.320300	3.166167	1.124434
H	-1.058170	3.203791	-0.638446
H	0.206706	-2.755723	-0.455794
H	-1.408772	-3.199649	-1.113545
H	-1.126084	-3.227126	0.646469
H	-5.063398	0.876214	0.548278
H	-5.074159	-0.910498	0.365214
H	-5.042719	0.139582	-1.067359
H	1.096841	0.245620	-3.275813
H	-0.249950	-0.769644	-2.666125
H	1.348437	-1.504061	-3.046442
H	5.055632	0.916343	-0.380847
H	5.068839	-0.830935	-0.682555
H	5.056359	-0.205997	1.000103

Table S5. Cartesian coordinates of PBE/TZ2P optimized triplet **2** (Å).

Atom	X	Y	Z
Fe	-0.003581	-0.001519	0.008657
N	1.292514	0.531576	1.262758
N	2.593847	0.482882	1.131310
N	1.296765	-0.559388	-1.230578
N	2.596058	-0.524454	-1.089105
N	-1.292276	1.366726	0.033063
N	-2.593877	1.227686	0.037856
N	-1.303716	-1.356182	-0.013203
N	-2.602423	-1.210018	-0.037847
C	3.170683	-0.021772	0.022743
C	4.679174	-0.008329	0.013346
C	0.915833	1.170481	2.535116
C	-0.927879	-2.778570	-0.094954
C	0.920659	-1.213083	-2.495965
C	-3.173522	0.012579	-0.010785
C	-0.911483	2.783829	0.160600
C	-4.681006	0.013470	-0.058845
H	1.168052	2.242835	2.519082
H	1.464081	0.699629	3.362759
H	-0.162427	1.052109	2.691302
H	0.164119	2.884207	-0.024292
H	-1.144433	3.158985	1.170005
H	-1.471180	3.385020	-0.569230

H	0.146262	-2.877909	0.098621
H	-1.158473	-3.182429	-1.093577
H	-1.493394	-3.355139	0.649998
H	-5.066805	0.978633	0.288187
H	-5.086699	-0.791619	0.567273
H	-5.043276	-0.152144	-1.085545
H	1.486809	-0.767882	-3.325663
H	-0.153634	-1.077547	-2.664827
H	1.152051	-2.289501	-2.457445
H	5.062389	0.776731	-0.656780
H	5.075791	-0.967512	-0.344232
H	5.054969	0.188424	1.023777

Table S6. Cartesian coordinates of PBE/TZ2P optimized quintet **2** (Å).

Atom	X	Y	Z
Fe	-0.001952	-0.011122	0.182376
N	1.287487	-0.011009	-1.322187
N	2.588814	0.002337	-1.251479
N	1.480158	-0.005358	1.455728
N	2.761382	0.007843	1.198519
N	-1.396436	-1.390347	0.095491
N	-2.677876	-1.215949	-0.083539
N	-1.372318	1.396316	0.073048
N	-2.656026	1.240253	-0.104316
C	3.239456	0.017854	-0.064722
C	4.745330	0.068136	-0.169136
C	0.817191	-0.048419	-2.713263
C	-1.002232	2.816661	0.142143
C	1.215574	-0.027017	2.901337
C	-3.225050	0.016152	-0.182759
C	-1.048984	-2.814950	0.188574
C	-4.723308	0.027850	-0.373523
H	1.123857	-0.989014	-3.196836
H	1.244678	0.789299	-3.284490
H	-0.277663	0.021757	-2.724733
H	0.038332	-2.913467	0.301103
H	-1.370989	-3.347191	-0.719466
H	-1.546756	-3.276143	1.055417
H	0.088339	2.900122	0.232051
H	-1.475489	3.294754	1.013550
H	-1.335472	3.342910	-0.765195
H	-5.045578	-0.876311	-0.903502

H	-5.028168	0.915352	-0.940828
H	-5.247092	0.053632	0.595282
H	1.711592	-0.890347	3.369772
H	0.132557	-0.094380	3.068101
H	1.596509	0.891208	3.374469
H	5.208638	-0.583650	0.582346
H	5.120960	1.089236	0.002964
H	5.061014	-0.244866	-1.170759

Table S7. Cartesian coordinates of PBE/TZ2P optimized singlet **3** (Å).

Atom	X	Y	Z
Fe	-0.000177	-0.000388	-0.017117
N	1.277444	1.086455	0.715754
N	2.590605	0.955027	0.734458
N	2.597445	-0.956515	-0.746657
N	1.283904	-1.085625	-0.742446
N	-1.278219	-1.087042	0.715222
N	-2.591344	-0.954960	0.734082
N	-2.597371	0.956421	-0.747275
N	-1.283804	1.084929	-0.743189
C	0.837909	2.233162	1.463957
C	1.541658	3.442691	1.369892
H	2.432959	3.487100	0.744903
C	1.086465	4.563693	2.059909
H	1.632314	5.504839	1.973666
C	-0.062601	4.489900	2.852876
H	-0.413298	5.369898	3.394281
C	-0.755668	3.281779	2.952664
H	-1.643726	3.208730	3.582407
C	-0.310434	2.153790	2.262633
H	-0.835817	1.203028	2.361509
C	3.183336	-0.001987	-0.001807
C	4.668177	-0.005348	0.010613
C	5.383526	0.831102	0.883933
H	4.832393	1.483191	1.561286
C	6.775762	0.820136	0.896166
H	7.308438	1.474552	1.590785
C	7.507706	-0.017914	0.042532
C	6.787592	-0.852299	-0.824806
H	7.329843	-1.519445	-1.499624
C	5.395376	-0.849883	-0.844914
H	4.853691	-1.503371	-1.528545

C	9.013852	-0.003818	0.039487
H	9.424696	-0.981131	-0.248864
H	9.415246	0.261511	1.027182
H	9.399304	0.737316	-0.679335
C	0.850215	-2.230928	-1.495916
C	-0.293034	-2.150217	-2.301791
H	-0.818170	-1.199381	-2.401770
C	-0.733671	-3.276912	-2.996927
H	-1.617583	-3.202759	-3.632332
C	-0.041223	-4.485209	-2.894765
H	-0.388305	-5.364219	-3.440099
C	1.102352	-4.560511	-2.094057
H	1.647369	-5.501939	-2.005627
C	1.553031	-3.440826	-1.399048
H	2.439684	-3.486589	-0.767629
C	-0.839207	-2.234362	1.462823
C	-1.543481	-3.443524	1.368045
H	-2.434907	-3.487130	0.743176
C	-1.088628	-4.565206	2.057192
H	-1.634851	-5.506078	1.970350
C	0.060603	-4.492441	2.850014
H	0.411080	-5.372988	3.390669
C	0.754139	-3.284656	2.950592
H	1.642332	-3.212414	3.580240
C	0.309246	-2.155994	2.261440
H	0.835059	-1.205528	2.360849
C	-3.183659	0.002301	-0.002220
C	-4.668487	0.006998	0.011049
C	-5.384109	-0.828542	0.885101
H	-4.833147	-1.480987	1.562256
C	-6.776277	-0.816502	0.897968
H	-7.309115	-1.470342	1.593018
C	-7.507986	0.021874	0.044326
C	-6.787653	0.855269	-0.823653
H	-7.329734	1.522461	-1.498548
C	-5.395394	0.851792	-0.844383
H	-4.853544	1.504440	-1.528678
C	-9.014146	0.009066	0.042413
H	-9.424227	0.981967	-0.261327
H	-9.415105	-0.239964	1.034582
H	-9.400792	-0.743456	-0.663820
C	-0.849337	2.229403	-1.497416
C	-1.551964	3.439608	-1.402677

H	-2.439347	3.486268	-0.772347
C	-1.100080	4.558476	-2.098266
H	-1.644955	5.500141	-2.011480
C	0.044580	4.482064	-2.897345
H	0.392649	5.360462	-3.443038
C	0.736747	3.273456	-2.997485
H	1.621426	3.198406	-3.631717
C	0.294794	2.147571	-2.301943
H	0.819638	1.196441	-2.400593

Table S8. Cartesian coordinates of PBE/TZ2P optimized quintet **3** (Å).

Atom	X	Y	Z
Fe	-0.000004	-0.002072	0.008823
N	1.390984	1.053659	0.957913
N	2.682429	0.945502	0.781253
N	2.674827	-0.957825	-0.781676
N	1.381571	-1.066415	-0.944101
N	-1.392295	-1.056716	0.956826
N	-2.683697	-0.946400	0.781012
N	-2.673665	0.957358	-0.781389
N	-1.380356	1.063677	-0.944646
C	1.015289	2.163542	1.768520
C	1.840882	3.292722	1.905604
H	2.798869	3.310032	1.387604
C	1.417834	4.367228	2.682415
H	2.059938	5.244794	2.777513
C	0.177158	4.336005	3.327824
H	-0.148901	5.183670	3.932354
C	-0.643869	3.214040	3.190373
H	-1.609997	3.174992	3.695792
C	-0.231202	2.132463	2.414757
H	-0.863432	1.245975	2.328224
C	3.237029	-0.004533	-0.004786
C	4.726862	-0.001735	-0.015180
C	5.457328	0.625361	1.008367
H	4.918771	1.110478	1.822480
C	6.850022	0.622518	0.996032
H	7.391604	1.108680	1.811528
C	7.571251	0.000919	-0.033286
C	6.838174	-0.626453	-1.050615
H	7.370261	-1.121376	-1.867066
C	5.445354	-0.630388	-1.046099

H	4.897343	-1.124095	-1.848683
C	9.077370	0.029587	-0.059238
H	9.487554	-0.852761	-0.569403
H	9.495850	0.066664	0.955973
H	9.445722	0.918447	-0.596840
C	0.996368	-2.183296	-1.740315
C	-0.254224	-2.154179	-2.378653
H	-0.881960	-1.263925	-2.298094
C	-0.676400	-3.242672	-3.139296
H	-1.645693	-3.205371	-3.638735
C	0.139328	-4.369422	-3.269552
H	-0.194183	-5.222551	-3.862251
C	1.384234	-4.398421	-2.632345
H	2.022229	-5.279550	-2.722061
C	1.816754	-3.317020	-1.870424
H	2.778196	-3.332302	-1.358754
C	-1.017635	-2.168636	1.765086
C	-1.844358	-3.297241	1.900035
H	-2.802501	-3.312489	1.382249
C	-1.422201	-4.373783	2.674519
H	-2.065173	-5.250890	2.767982
C	-0.181310	-4.345186	3.319628
H	0.144046	-5.194462	3.922279
C	0.640837	-3.223772	3.184317
H	1.607132	-3.186767	3.689576
C	0.229047	-2.140157	2.411088
H	0.862124	-1.254091	2.326272
C	-3.237105	0.005227	-0.003915
C	-4.726947	0.005193	-0.013121
C	-5.457686	-0.621011	1.010777
H	-4.919331	-1.107295	1.824329
C	-6.850382	-0.615957	0.999432
H	-7.392160	-1.101528	1.815149
C	-7.571351	0.007070	-0.029206
C	-6.837999	0.633651	-1.046825
H	-7.369873	1.129671	-1.862748
C	-5.445170	0.635383	-1.043293
H	-4.896940	1.128444	-1.846122
C	-9.077529	-0.019317	-0.054152
H	-9.486730	0.863896	-0.563644
H	-9.495389	-0.056227	0.961322
H	-9.447587	-0.907367	-0.591916
C	-0.993805	2.178774	-1.742725

C	-1.812444	3.313604	-1.874272
H	-2.773605	3.331271	-1.362153
C	-1.378611	4.393020	-2.638285
H	-2.015238	5.275021	-2.729136
C	-0.134062	4.360942	-3.276080
H	0.200499	5.212536	-3.870390
C	0.679919	3.233138	-3.144327
H	1.648913	3.193456	-3.644164
C	0.256438	2.146646	-2.381564
H	0.882801	1.255552	-2.299820

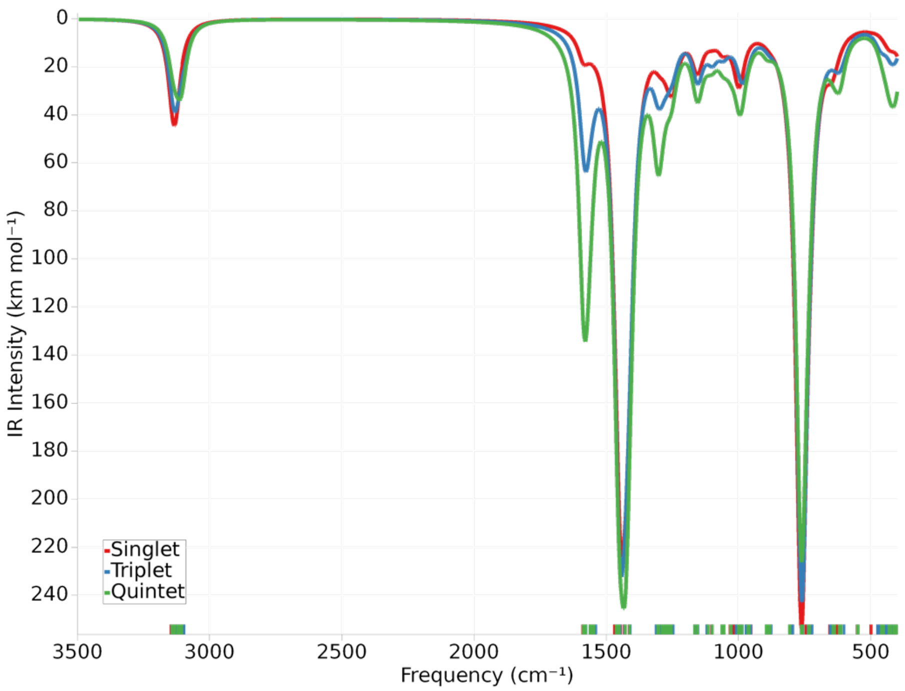

Figure S1. Calculated IR spectrum for the singlet, triplet, and quintet spin states of **1**.

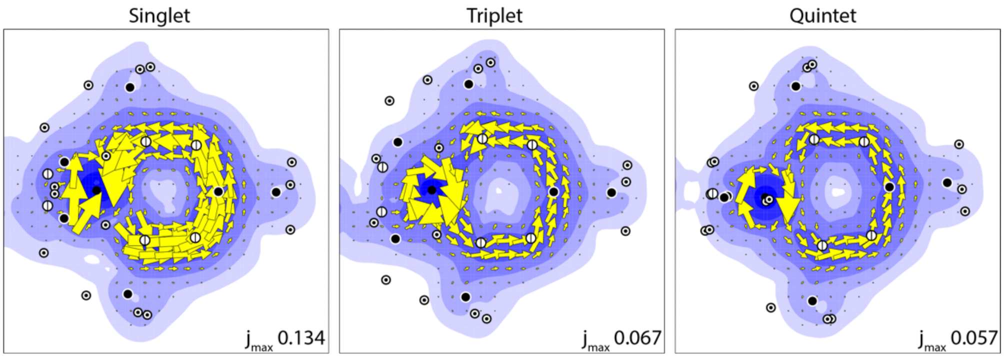

Figure S2. Plots of the induced current π-density using PBE0 for the different spin states of **2**.
